# Influence of Reaction Conditions on Enzymatic Enantiopreference: the Curious Case of HEwT in the Synthesis of THF‐Amine

**DOI:** 10.1002/cbic.202200335

**Published:** 2022-07-01

**Authors:** Christian M. Heckmann, Lucia Robustini, Francesca Paradisi

**Affiliations:** ^1^ School of Chemistry University of Nottingham University Park Nottingham NG7 2RD UK; ^2^ Department of Chemistry Biochemistry and Pharmaceutical Sciences University of Bern Freiestrasse 3 3012 Bern Switzerland

**Keywords:** amines, biocatalysis, enantioselectivity, enzyme, THF ketones, transaminases

## Abstract

Enzymatic enantiopreference is one of the key advantages of biocatalysis. While exploring the synthesis of small cyclic (chiral amines) such as 3‐aminotetrahydrofuran (THF‐amine), using the (*S*)‐selective transaminase from *Halomonas elongata* (HEwT), inversion of the enantiopreference was observed at increasing substrate loadings. In addition, the enantiopreference could be altered by variation of the ionic strength, or of the co‐solvent content in the reaction mixture. For example, using otherwise identical reaction conditions, the presence of 2 M sodium chloride gave (*R*)‐THF‐amine (14 % *ee*), while the addition of 2.2 M isopropyl alcohol gave the (*S*)‐enantiomer in 30 % *ee*. While the underlying cause is not currently understood, it appears likely that subtle changes in the structure of the enzyme cause the shift in enantiopreference and are worth exploring further.

## Graphical abstract

**While scaling‐up** the transamination of THF‐ketone, an unusual switch in enantioselectivity was observed which correlated with increasing concentrations of amine donor. At high concentration of organic molecules, the enzyme was (*S*)‐selective, while at high ionic strength the enzyme became (*R*)‐selective. The mechanism for this effect is not yet clear.

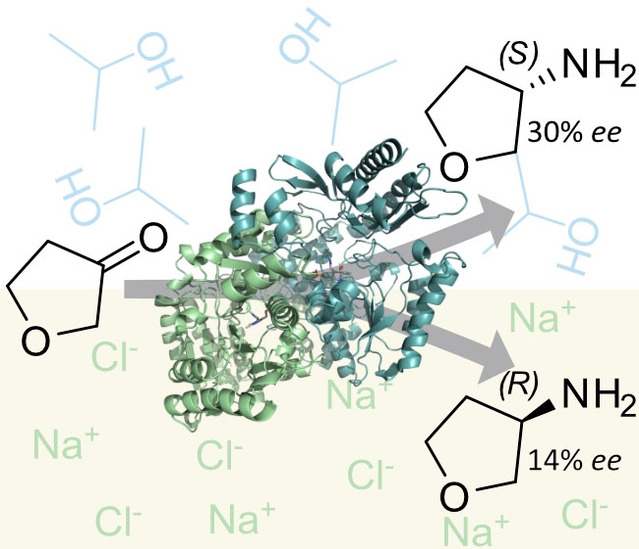

Enzymes are often highly enantioselective, which makes them attractive catalysts in organic synthesis, in particular for the pharmaceutical industry where high enantiopurity is required.[^1^, ^2^] For example, in the synthesis of chiral amines (featured in many active pharmaceutical ingredients) chemical catalysts can often only reach low *enantiomeric excess* (*ee*), in particular in the case of aliphatic amines.^[3]^ On the other hand, there are a variety of classes of enzymes capable of synthesising chiral amines with excellent enantioselectivity.[^2^, ^4^, ^5^] One major class of these enzymes are ω‐transaminases (TAs), which catalyse the formal reductive amination of pro‐chiral ketones using a sacrificial amine donor, which is oxidized to the carbonyl.^[6]^ TAs are usually highly enantioselective (frequently only one enantiomer is detectable),[^7^, ^8^, ^9^] the selectivity being due to steric discrimination, conferred by a small and a large binding pocket.^[6]^ In previous work (Scheme 1),^[10]^ the synthesis of four aliphatic cyclic amines was investigated, where steric discrimination of either side of the pro‐chiral ketone is challenging. Indeed, only moderate *ee* values were obtained. Curiously, the *ee* values decreased with two of the four tested substrates when employing the (S)‐selective transaminase from *Halomonas elongata* (HEwT) in flow rather than in batch conditions.^[10]^ Low enantioselectivity was also noted when HEwT was challenged with butanone.^[11]^ Here, this behaviour of HEwT is further investigated, focussing on tetrahydrofuran‐3‐one (THF‐ketone) as the substrate (Scheme 1).

While exploring the intensification of this reaction using HEwT (10, 100, and 300 mM scale with 5 eq. of isopropylamine (IPA) as the amine donor), different *ee* values were obtained at the different substrate concentrations, as seen in Table 1, while conversions decreased slightly with increasing concentrations of THF‐ketone. Notably, the enantiopreference of the enzyme changed from (*S*) to (*R*) at the highest concentration.

**Table 1:** Intensification of biotransformations of 3‐amino‐THF using HEwT. Reactions on a 10, 100 or 300 mM scale, containing HEwT (lyophilized cell‐free extract, 50 mg/mmol), IPA (5 eq.), PLP (1 mM), KP_*i*_‐buffer (50 mM), and DMSO (10 %); pH 8. Reactions were incubated at 30 °C for 48 h.

Scale (mM)	Conversion (%)^[a]^	*ee* ^[b]^ (%)
10	82±3	11 (*S*)
100	76±1	4 (*R*)
300	54±2	17 (*R*)

[a] Conversion determined by RP‐HPLC, following the production of THF‐amine (after FMOC derivatization), using a calibration curve ±1 standard deviation (n=2). [b] *ee* determined by chiral GC‐FID following acetylation.

In light of this unusual result, also in contrast to those obtained previously (Scheme 1),^[10]^ attempts were made to better understand this variability and switching behaviour. An initial hypothesis was postulated whereby different conditions in the experiments (catalyst loading, reaction temperature and time, immobilized or soluble enzyme, and substrate loading) could explain the differences in *ee* through thermodynamic vs kinetic control;^[12]^ in particular, longer reaction times or higher catalyst loadings (or a more stable (e. g. immobilized) catalyst) would favour a thermodynamic outcome, *i. e*. the racemate (Scheme 2). Such behaviour has been previously reported with chiral organocatalysts.^[13]^

**Scheme 1:**
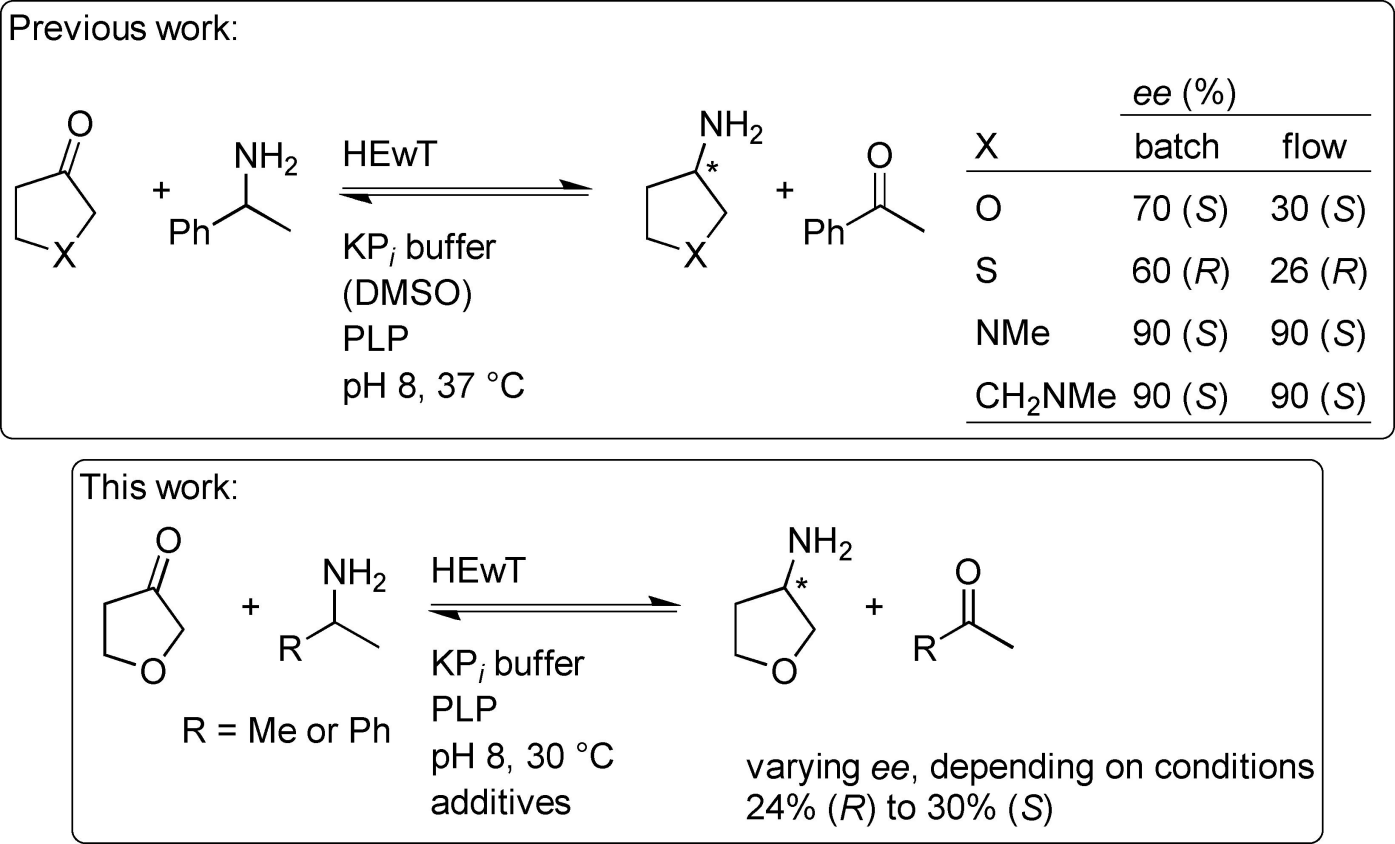
Previous work:^[10]^ synthesis of THF‐amine, as well as 3‐aminotetrahydrothiophene, *N*‐methyl‐3‐aminopyrrolidine, and *N*‐methyl‐3‐aminopiperidine, showing variable *ee* values in batch vs. flow. Current work: Synthesis of THF‐amine from THF‐ketone, using the (*S*)‐selective TA HEwT, with variable *ee* values in batch depending on the reaction conditions.

**Scheme 2:**
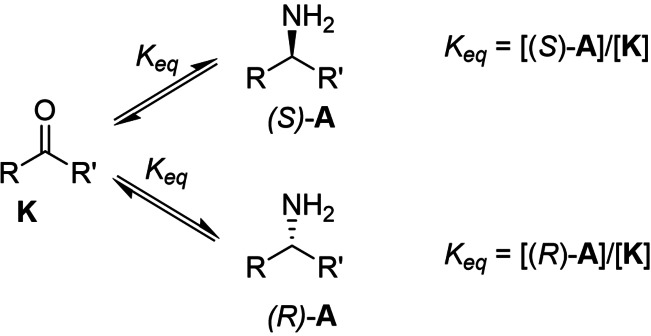
Simplified equilibrium for a generic reductive amination. Since both enantiomers have identical thermodynamic properties (standard Gibbs free energy), the equilibrium constants between the prochiral ketone (**K**) and either enantiomer of the amine (**A**) are identical. Since both equations have the same concentration of ketone in the denominator, the concentration of each enantiomer has to be identical for both equilibria to be satisfied.

To observe the thermodynamic effects, the *ee* of a reaction was monitored over time. Additionally, the *ee* of a series of reactions with increasing enzyme concentrations was also studied. To rule out effects of other enzymes present in the crude, purified HEwT (IMAC) was used. Highest *ee* values were expected at the shortest reaction times and with the lowest enzyme concentration and should decrease as either time or enzyme concentration increases. As can be seen in Figure 1A−C, this behaviour is in fact observed, however the maximum *ee* values obtained under those conditions were approx. 16 % (*S*), and thus it seemed unlikely that this effect alone was strong enough to explain the range of *ee* values previously observed. Indeed, this effect would also not explain the concentration‐dependent switching behaviour that is also observed with the purified enzyme (Figure 1D), ruling out interference from other enzymes in the crude.

**Figure 1:**
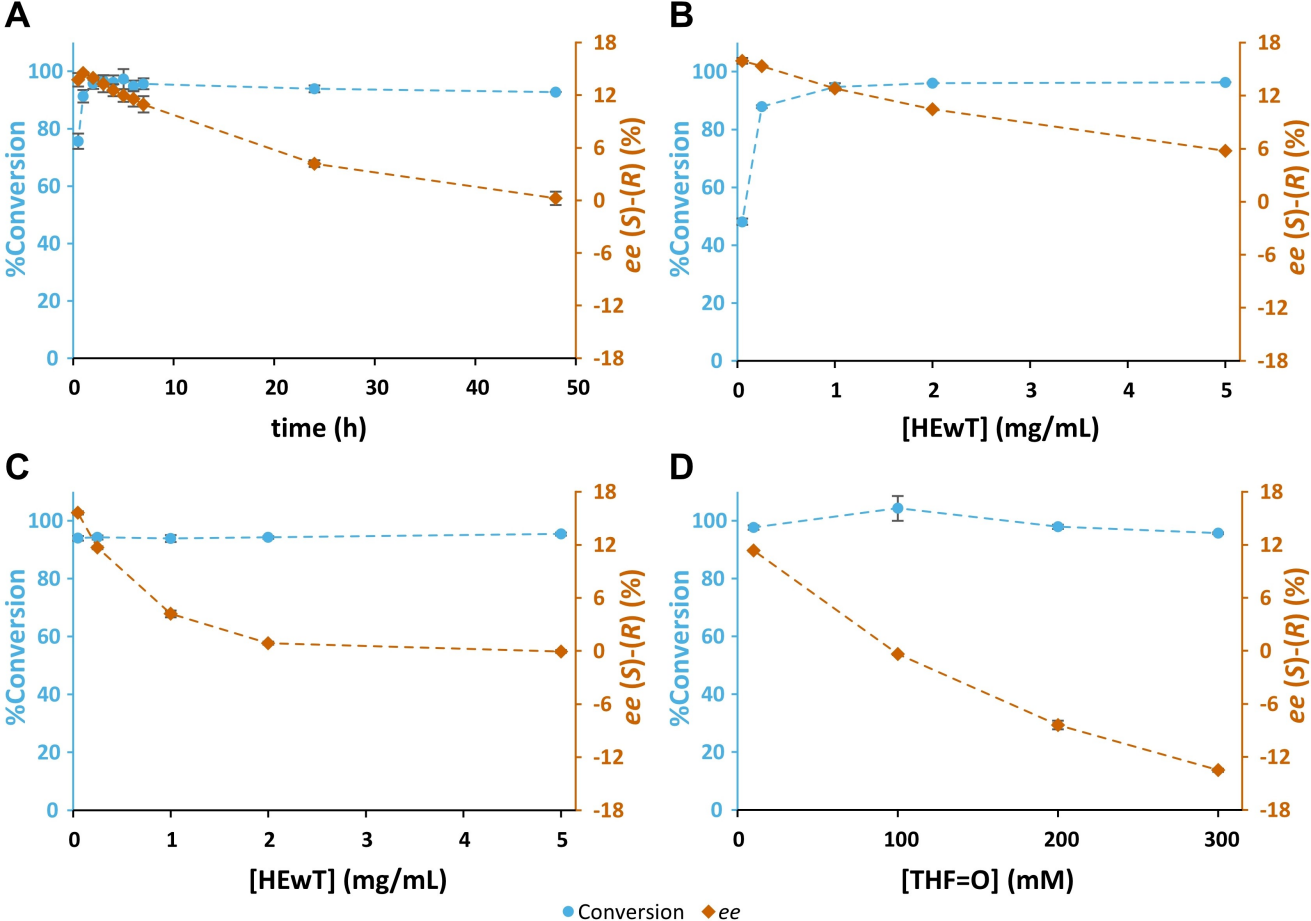
A: The production of THF‐amine from THF‐ketone over time, in a biotransformation containing THF‐ketone (10 mM), (*S*)‐α‐methylbenzylamine (SMBA) (1 eq.) and purified HEwT (1.0 mg/mL). B: The production of THF‐amine from THF‐ketone with increasing concentrations of purified HEwT, in biotransformations containing THF‐ketone (10 mM) and SMBA (1 eq.). Samples taken after 3 h and C: samples taken after 24 h. D: The production of THF‐amine from THF‐ketone (THF=O) at increasing substrate concentrations, in biotransformations containing IPA (5 eq.), and purified HEwT (0.72 mg/mL). Samples taken after 3 h. All reactions contained PLP (1 mM), KP_*i*_‐buffer (100 mM); pH 8, 30 °C. Conversions determined by chiral RP‐HPLC, after FMOC‐derivatization. Signed *ee* values; +ve (*S*)‐enantiomer, ‐ve (*R*)‐enantiomer. Connecting lines added for clarity.

Having ruled out thermodynamic effects and the presence of other enzymes in the crude as causes for this behaviour, the focus was shifted on how the reaction environment affects the enantioselectivity of the enzyme. It has previously been reported that when using enzymes in organic media the nature of the organic solvent and its water content can affect the enantioselectivity of lipases and proteases, and even lead to an inversion in enantioselectivity.[^14^, ^15^] To verify whether the behaviour observed with HEwT was caused by an increase in concentration of either or both substrates, biotransformations were set up containing fixed concentrations of either THF‐ketone or IPA, with varying amounts of the other substrate. As can be seen in Figure 2A, increasing the concentration of THF‐ketone from 10 to 300 mM (IPA fixed at 50 mM), the enzyme remains (*S*)‐selective, with slight variation. However, when IPA was varied from 50 to 1500 mM (THF‐ketone fixed at 100 mM), the same switching behaviour was observed as when both were varied proportionally (compare Figure 1D and Figure 2B).

**Figure 2:**
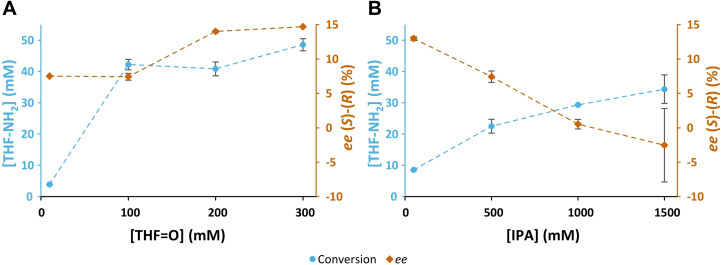
A: The production of THF‐amine from THF‐ketone (THF=O) at increasing concentration of THF‐ketone, in biotransformations containing a fixed concentration of IPA (50 mM), and purified HEwT (1 mg/mL). Samples taken after 3 h. B: The production of THF‐amine from THF‐ketone (THF=O) at increasing concentration of IPA, in biotransformations containing a fixed concentration of THF‐ketone (100 mM), and purified HEwT (1 mg/mL). Samples taken after 3 h. All reactions contained PLP (1 mM), KP_*i*_‐buffer (50 mM); pH 8, 30 °C. Conversions determined by RP‐HPLC, *ee* values determined by chiral RP‐HPLC, after FMOC‐derivatization. Error bars represent standard deviations (n=2). Signed *ee* values; +ve (*S*)‐enantiomer, ‐ve (*R*)‐enantiomer. Connecting lines added for clarity.

Previous docking studies^[10]^ suggested that the enantioselectivity of HEwT could be explained for this substrate via hydrogen bonding of the ring oxygen to W56 as the substrate enters the active site (Figure 3). From this docking, it appears that the neighbouring methylene (C5) is facing the entrance of the active site and could possibly interact with the solvent. Thus, an initial hypothesis was postulated that competing hydrogen bonding of THF‐ketone to the reaction medium (*i. e*. with IPA, which at pH 8 is protonated (p*K*_a_ 10.63)^[16]^) might favour the substrate entering the active site “C5‐first” rather than ”oxygen‐first,” favouring the production of (*R*)‐THF‐amine. However, other effects pertinent to the enzyme are also possible, *e. g*. the increasing organic content (decreasing water content) or increasing ionic strength might result in structural changes of the enzyme that favour the production of the (*R*)‐enantiomer.

**Figure 3:**
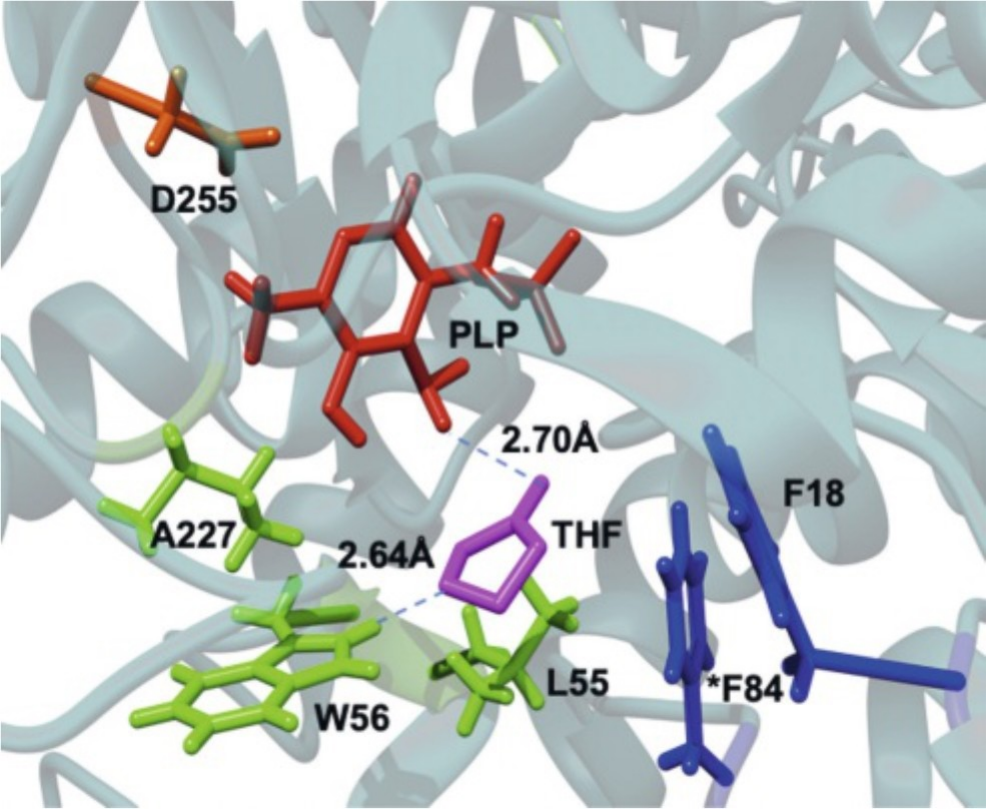
Docking of THF‐ketone into the entrance of the active site of wild‐type HEwT, showing the hydrogen bond to W56. Figure reproduced from ref. [10] (CC BY 4.0 license).

To separate the different effects of increasing IPA concentrations (i. e. hydrogen bonding, organic content, and ionic strength), experiments were set up using (*S*)‐methylbenzylamine (SMBA) as the amine donor with varying concentrations of the following additives: isopropyl alcohol (a weaker hydrogen bond donor than (protonated) IPA, with similar size and structure), sodium chloride (increasing the ionic strength without altering the concentration of hydrogen bond donors or the organic content), and ammonium chloride (which, despite its lower p*K*_a_ of 9.21, is also protonated at pH 8 and should provide a similar hydrogen bond donor capacity to IPA, whilst not increasing the organic content).

Thus, if the switch in enantiopreference is indeed due to competing hydrogen bonding to the reaction medium, a similar effect should be observed for ammonium chloride and to a lesser extent isopropyl alcohol. However, if it is due to increasing organic content which affects the structure of the enzyme, it should be observed only with isopropyl alcohol; and if it is due to increasing ionic strength it should be observed only with sodium chloride and ammonium chloride. As can be seen in Figure 4A–C, increasing the isopropyl alcohol concentration does indeed affect the *ee*, but in the opposite direction to what was expected. On the other hand, increasing concentrations of ammonium chloride and sodium chloride switches the enantioselectivity from (*S*) to (*R*).

**Figure 4:**
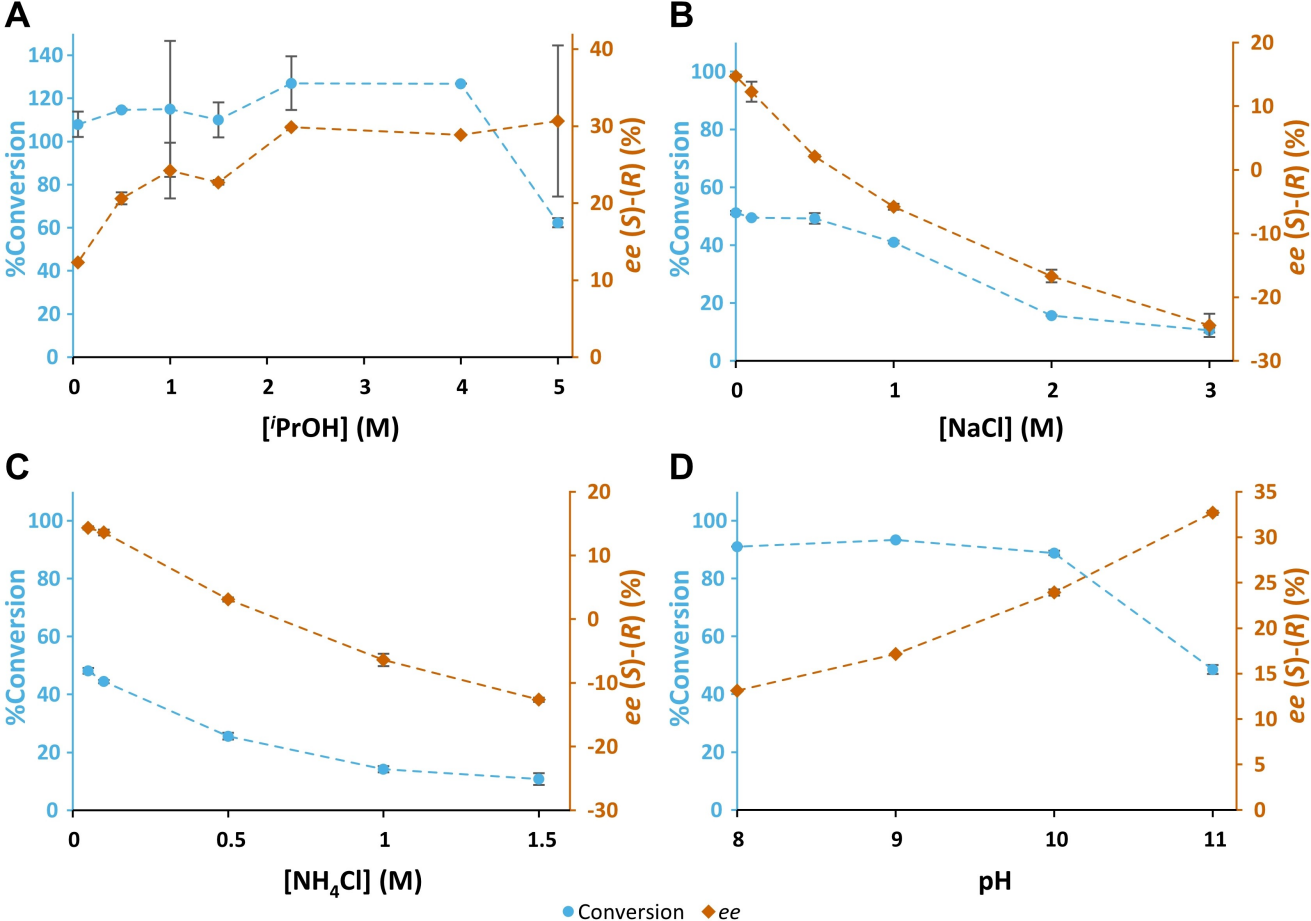
A−C: The production of THF‐amine from THF‐ketone (THF=O) in biotransformations containing THF‐ketone (10 mM), SMBA (1 eq.), purified HEwT (0.25 mg/mL), PLP (0.1 mM), and KP_*i*_‐buffer (50 mM); pH 8, with varying concentrations of additives. Samples taken after 3 h. A: ^*i*^PrOH (0.05 to 5 M), B: NaCl (0.01 to 3 M)), C: NH_4_Cl (0.05 to 1.5 M)). In the case of C, the decrease of conversion at increasing concentrations of ammonium chloride might be (partially) due to interference of ammonium in the FMOC derivatization, *i. e*. an artefact of the analysis rather than a real reduction in conversion. This is supported by the complete consumption of FMOC−Cl, the appearance of an unidentified peak (presumably FMOC‐NH_3_), and a reduction in size of the FMOC‐derivatized SMBA peak (Figure S1). D: The production of THF‐amine from THF‐ketone (THF=O) in biotransformations containing THF‐ketone (10 mM), SMBA (1 eq.), purified HEwT (0.25 mg/mL), PLP (1 mM), and KP_*i*_‐buffer (100 mM), with varying pH. Samples taken after 3 h. All reactions carried out at 30 °C. Conversions (A−C) determined by RP‐HPLC, conversions (D) and *ee* values determined by chiral RP‐HPLC, after FMOC‐derivatization. Error bars represent standard deviations (n=2). Signed *ee* values; +ve (*S*)‐enantiomer, ‐ve (*R*)‐enantiomer. Connecting lines added for clarity.

This suggests that the switch in enantioselectivity may be predominantly caused by subtle structural changes of the enzyme, with a more (*S*)‐selective structure dominating in hydrophobic/organic reaction media and a more (*R*)‐selective structure in hydrophilic reaction media. Increasing the pH (Figure 4D) also enhances the (*S*)‐selectivity of HEwT, which may be due to lower amounts of protonated amines, rendering the reaction medium more hydrophobic while lowering the ionic strength. However, in the absence of direct evidence of such structural changes, this is not a definitive conclusion, and it might indeed be the case that a more hydrophobic environment favours THF‐ketone entering the active site “oxygen‐first” and a more hydrophilic environment favours it entering “C5‐first.” Increasing ionic strength might also disrupt the hydrogen bonding to W56 or alter the solvation sphere around THF‐ketone in solution. Such solvation effects play a crucial role in the stereoselectivity of chemical reactions.^[17]^ Indeed, all three mechanisms (structural effects on the enzyme, binding of solvent into the active site and solvation of the substrate) have previously been proposed to affect the stereoselectivity of chymotrypsin in organic media.

Next, the role of the structure of the amino donor and amino acceptor was investigated. As can be seen above, the switch in enantiopreference can be observed with either IPA or SMBA as amino donor. However, due to substrate inhibition by SMBA,^[18]^ increasing its concentration while keeping THF‐ketone constant (analogous to the experiment with IPA shown in Figure 2A) was no feasible. Thus, the smart amine donor cadaverine^[19]^ was chosen. As shown in Figure 5A, increasing concentrations of cadaverine indeed decreased the (*S*)‐selectivity of the enzyme; however, no switch in enantioselectivity was observed. It should be noted, however, that only concentrations of up to 250 mM could be tried, which is below the range where a switch in enantioselectivity was observed with IPA.

**Figure 5:**
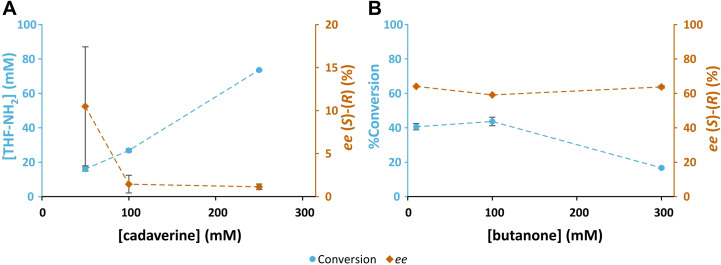
A: The production of THF‐amine from THF‐ketone (THF=O) at increasing concentrations of cadaverine, in biotransformations containing a fixed concentration of THF‐ketone (100 mM), and purified HEwT (1 mg/mL). Samples taken after 3 h. B: The production of 2‐aminobutane from butanone at increasing substrate concentrations, in biotransformations containing IPA (5 eq.), and purified HEwT (1 mg/mL). Samples taken after 3 h. All reactions contained PLP (0.1 mM), KP_*i*_‐buffer (50 mM); pH 8, 30 °C. Conversions determined by RP‐HPLC, *ee* values determined by chiral RP‐HPLC, after FMOC‐derivatization. Error bars represent standard deviations (n=2). Signed *ee* values; +ve (*S*)‐enantiomer, ‐ve (*R*)‐enantiomer. Connecting lines added for clarity.

In previous work,^[11]^ a slight decrease in *ee* with increasing substrate concentration had been observed with butanone. In light of the results with THF‐ketone this was explored further. However, as can be seen in Figure 5B, the *ee* only varied slightly at higher substrate and IPA concentrations (in contrast to the experiment with THF‐ketone shown in Figure 1D). Thus, the effect observed with THF‐ketone is not observed with butanone. This is consistent with the enantioselectivity for butanone, unlike for THF‐ketone, which is primarily determined by sterics. Indeed, the effect previously observed coincided with increasing conversions due to higher concentration of enzyme and can thus be fully explained by thermodynamic effects.^[11]^

Finally, the influence of the nature of the co‐solvents was investigated. Two protic solvents and two aprotic solvents were chosen. As can be seen in Figure 6, increasing log *P* correlates with enhanced (*S*)‐selectivity, consistent with previous observations. On the other hand, neither the solvent excluded volume, nor the protic/aprotic nature of the solvent showed any clear trend. Rother and co‐workers^[20]^ reported that for carboligations catalysed by thiamine diphosphate dependent enzymes, decreasing the size of the co‐solvent correlates with decreased (*S*)‐selectivity and even inversion to (*R*)‐selectivity. This was viewed as evidence that the smaller solvent molecules can block the binding pocket for these enzymes, favouring binding of the substrate in a flipped orientation. The lack of such size‐dependency in the present case seems to preclude such a direct binding to the active site in the present case.

**Figure 6:**
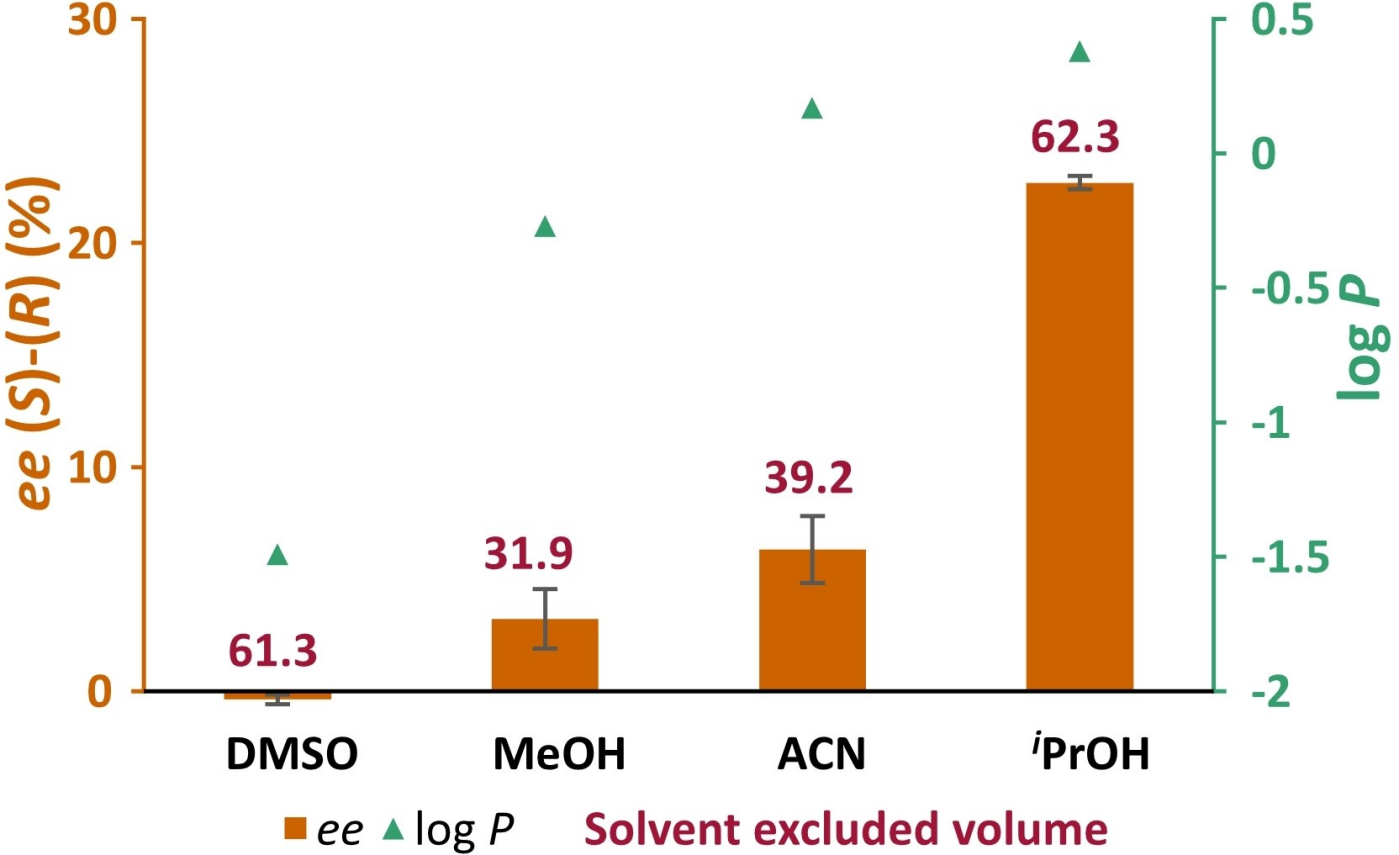
Effect of different co‐solvents on the enantioselectivity of HEwT for the production of THF amine from THF‐ketone (THF=O) in biotransformations containing THF‐ketone (10 mM), SMBA (1 eq.), purified HEwT (0.25 mg/mL), PLP (0.1 mM) in KP_*i*_‐buffer (50 mM); pH 8, 30 °C with varying co‐solvents (1.5 M). Samples taken after 3 h. The *ee* values determined by chiral RP‐HPLC, after FMOC‐derivatization. Error bars represent standard deviations (n=2). Signed *ee* values; +ve (S)‐enantiomer, ‐ve (R)‐enantiomer. Also shown are the log *P* and Conolly solvent excluded volume of each solvent, as reported by ChemDraw v. 20.0.

To summarize, attempting to intensify the previously reported synthesis of THF‐amine using HEwT, a curious switching in enantiopreference was observed. Initially this was noted at increasing substrate loading, it was then shown to be caused by an increase in ionic strength. Further experiments showed that increasing the hydrophobicity of the reaction increases the (*S*)‐selectivity of the enzyme, while increasing the ionic strength enhances the (*R*)‐selectivity. The effect does not appear to depend on the size of the “additive”, making a direct binding to the active site an unlikely explanation. Thus, the effect is most likely due to structural changes in the enzyme under these varying conditions, i. e. increasing the ionic strength of the medium is expected to increase the strength of hydrophobic interactions within the protein structure, while increasing the hydrophobicity of the medium would decrease those interactions, subtly affecting the structure. However, if the enantioselectivity of HEwT is indeed determined by the orientation in which the substrate enters the active site (as previously postulated), a direct effect of the reaction medium on that orientation due to solvation effects on the substrate may also be possible. A similar effect could not be observed with butanone, a substrate where the enantioselectivity is determined by steric discrimination provided by a small and large binding pocket.

Clearly, further studies are needed, and may include obtaining structures of the enzyme under varying ionic strengths/organic content, circular dichroism spectroscopy, and point mutations of the W56 residue to confirm its role in the enantiopreference of HEwT for that substrate. Additionally, molecular dynamic simulations might offer additional insight into the trajectory and interactions with solvent/residues of THF‐ketone as it enters the active site.

## Conflict of interest

The authors declare no conflict of interest.

## Supporting information

As a service to our authors and readers, this journal provides supporting information supplied by the authors. Such materials are peer reviewed and may be re‐organized for online delivery, but are not copy‐edited or typeset. Technical support issues arising from supporting information (other than missing files) should be addressed to the authors.

Supporting Information

## Data Availability

The data that support the findings of this study are available from the corresponding author upon reasonable request.

## References

[1] B. Hauer , ACS Catal. 2020, 10, 8418–8427.

[2] M. Breuer , K. Ditrich , T. Habicher , B. Hauer , M. Keßeler , R. Stürmer , T. Zelinski , Angew. Chem. Int. Ed. 2004, 43, 788–824; 10.1002/anie.200300599 14767950

[3] T. Ghosh , M. Ernst , A. S. K. Hashmi , T. Schaub , Eur. J. Org. Chem. 2020, 4796–4800.

[4] M. D. Patil , G. Grogan , A. Bommarius , H. Yun , ACS Catal. 2018, 8, 10985–11015.

[5] D. Ghislieri , N. J. Turner , Top. Catal. 2014, 57, 284–300.

[6] I. Slabu , J. L. Galman , R. C. Lloyd , N. J. Turner , ACS Catal. 2017, 7, 8263–8284.

[7] F. G. Mutti , C. S. Fuchs , D. Pressnitz , N. G. Turrini , J. H. Sattler , A. Lerchner , A. Skerra , W. Kroutil , Eur. J. Org. Chem. 2012, 1003–1007.

[8] F. G. Mutti , C. S. Fuchs , D. Pressnitz , J. H. Sattler , W. Kroutil , Adv. Synth. Catal. 2011, 353, 3227–3233.

[9] C. K. Savile , J. M. Janey , E. C. Mundorff , J. C. Moore , S. Tam , W. R. Jarvis , J. C. Colbeck , A. Krebber , F. J. Fleitz , J. Brands , P. N. Devine , G. W. Huisman , G. J. Hughes , Science 2010, 329, 305–310. 20558668 10.1126/science.1188934

[10] E. Hegarty , F. Paradisi , Chimia 2020, 74, 890–894. 33243325 10.2533/chimia.2020.890

[11] C. M. Heckmann , B. Dominguez , F. Paradisi , ACS Sustainable Chem. Eng. 2021, 9, 4122–4129.

[12] S. R. Marsden , L. Mestrom , D. G. G. McMillan , U. Hanefeld , ChemCatChem 2020, 12, 426–437.

[13] G. Rulli , N. Duangdee , K. Baer , W. Hummel , A. Berkessel , H. Gröger , Angew. Chem. Int. Ed. 2011, 50, 7944–7947; 10.1002/anie.201008042 21744441

[14] G. Carrea , G. Ottolina , S. Riva , Trends Biotechnol. 1995, 13, 63–70.

[15] K. Kawashiro , H. Sugahara , S. Sugiyama , H. Hayashi , Biotechnol. Lett. 1995, 17, 1161–1166.

[16] H. K. Hall , J. Am. Chem. Soc. 1964, 86, 5709.

[17] G. Cainelli , P. Galletti , D. Giacomini , Chem. Soc. Rev. 2009, 38, 990–1001. 19421577 10.1039/b802815j

[18] L. Cerioli , M. Planchestainer , J. Cassidy , D. Tessaro , F. Paradisi , J. Mol. Catal. B 2015, 120, 141–150.

[19] A. Gomm , W. Lewis , A. P. Green , E. O'Reilly , Chem. Eur. J. 2016, 22, 12692–12695. 27411957 10.1002/chem.201603188

[20] T. Gerhards , U. MacKfeld , M. Bocola , E. Von Lieres , W. Wiechert , M. Pohl , D. Rother , Adv. Synth. Catal. 2012, 354, 2805–2820. 23349644 10.1002/adsc.201200284 PMC3549479

