# Supplementary material for: Influence of Reaction Conditions on Enzymatic Enantiopreference: the Curious Case of HEwT in the Synthesis of THF‐Amine
Source: Chembiochem. 2022 Jul 1;23(15):e202200335. doi: 10.1002/cbic.202200335 (PMC9400895; doi:10.1002/cbic.202200335)
Supplement: Supplementary file 1 — Supporting Information [file CBIC-23-0-s001.pdf]

ChemBioChem

Supporting Information

Influence of Reaction Conditions on Enzymatic Enantiopreference: the Curious Case of HEwT in the Synthesis of THF-Amine

Christian M. Heckmann[+], Lucia Robustini[+], and Francesca Paradisi*

Expression of HEwT

E. coli BL21 (DE3) or BL21 STAR™ (DE3) were transformed with pMP89a–HEwT and grown on selective LB–agar plates (amp 100 µg/mL) at 37 °C overnight. ZYP–AI medium[1] (300 mL; amp 100 µg/mL) was inoculated with a single colony of transformed *E. coli* and incubated for 20 h at 37 °C, 180 rpm (19 mm throw). Cells were harvested (4500 g, 20 min, 4 °C) and stored at –20 °C either as pellets or in loading buffer.

Enzyme purification

HEwT was purified using the Äkta Start system: Pellets were resuspended in loading buffer (potassium phosphate (50 mM), sodium chloride (100 mM), imidazole (30 mM), PLP (0.1 mM), pH 8.0; 3:1 *v:w*), lysed on ice by sonication ($^1/_4$″ probe, 50% amplitude, 5 s on, 5 s off, for 12 min), and cell debris was removed by centrifugation (25,500 g, 4 °C, 60 min). The filtered (0.45 µm) supernatant was loaded (1 mL/min) onto a Qiagen Ni-NTA Superflow column (5 mL), washed with loading buffer (4 CV), followed by a step with 10% elution buffer (loading buffer containing imidazole (300 mM); 5 CV). The protein was then eluted with 100% elution buffer in fractions of 2.5 mL. Fractions containing protein were combined and dialysed against ice–cold dialysis buffer (potassium phosphate (50 mM), PLP (0.1 mM), pH 8.0; 800 mL); the buffer was renewed after the first 2 hours of dialysis. Enzyme concentration was estimated by the absorbance at 280 nm (non-denatured protein), using predicted extinction coefficients (54214.57 Da, ε = 62340 M^{-1} cm^{-1}https://web.expasy.org/protparam/). Protein samples were stored at 4 °C until further use.

Activity assays

Activity assays were based on the method by Schätzle *et al.*[9] as applied in Cerioli *et al.*[10], in UV-free 96-well plates using the EPOCH 2 plate reader at 30 °C, following the production of acetophenone from SMBA at 245 nm (pathlength 0.84 cm, calculated according to $\frac{A_{977}-A_{900}}{0.18}$); ε = 12.6 mM^{-1} cm^{-1}).

Biotransformations

Reactions were set up containing ketone (10-300 mM), amine donor (SMBA or isopropylamine in varying amounts as indicated), PLP (0.1-1 mM), DMSO (0-11 % (*v/v*)) and HEwT (either lyophilized crude or purified), and additives (were indicated), in potassium phosphate buffer (50-100 mM), pH 8. Tetrahydrofuran-3-one was added from a 10-fold concentrated stock in buffer, IPA was added from a pH adjusted (HCl) stock in buffer, and SMBA was added from a 10-fold concentrated pH adjusted (HCl) stock in buffer containing 10% DMSO. Additives (*i*PrOH, ammonium chloride, sodium chloride) were added from pH adjusted (HCl) stocks in buffer. Reactions were incubated at 30 or 37 °C for the indicated amount of time. Conversions and enantiomeric excess were determined either by extraction

into ethyl acetate (200 µL NaOH (5 M), then EtOAc (2×500 µL)) , acetylation (Et_3N (20 µL) and Ac_2O (20 µL)), and analysis by GC-FID, based on the relative peak areas of SMBA and acetophenone; or by FMOC-derivatization (see below) and analysis by chiral RP-HPLC, determining the concentration of product using a calibration curve (as indicated). When butanone was used as amino acceptor, the reaction set up contained ketone (10-300 mM), IPA as amino donor (either 100 mM or 5 eq., depending on the experiment) PLP (0.1 %), DMSO (5% v/v) and HeWT (1 mg/mL) resuspended in potassium phosphate buffer 50 mM pH8. When cadaverine was used ad aminodonor, instead of IPA, the reaction set up was as following: tetrahydrofuran-3-one (100 mM), cadaverine (50-250 mM), PLP (0.1 Mm), HEwT (1 mg/mL) in in potassium phosphate buffer 50 mM pH8. Due to it diamine structure, cadaverine pushes the pH of the reaction above the operational limit of the enzyme (>11 pH), reason why a more concentrated stock (2 M) was prepared in potassium phosphate buffer 1 M pH8. The detection of butylamine and the amine product of cadaverine assay, was detected at UV-VIS upon Fmoc- derivatisation by RP-HPLC.

FMOC derivatization

Borate buffer (100 mM, pH 9; 200 µL), the sample to be analysed (diluted to a total amine concentration not exceeding 25 mM; 100 µL), and FMOC–Cl (15 mM, in acetonitrile; 400 µL) were combined and mixed by vortexing. 200 µL of that mixture were added to 800 µL of hydrochloric acid (0.2%) and acetonitrile (1:1 v/v) and analysed by reverse–phase HPLC.

Chromatography

GC-FID: Thermo Scientific™ Trace™ 1310 GC equipped with an Agilent CHIRASIL-DEX CB (25 m x 0.25 mm x 0.25 µm) column: 0 min 40 °C, 2 min 40 °C, 7.5 min 150 °C, 12.5 min 150 °C, 14.167 min 200 °C, 18.167 min 200 °C. Injector temperature 230 °C, split ratios 1:10 to 1:100, continuous flow 1.7 mL/min, FID temperature 250 °C, injection volume 1 µL. Helium was used as carrier gas. Retention times in min (amines acetylated): (*S*)-tetrahydrofuran-3-amine (9.6), (*R*)-tetrahydrofuran-3-amine (9.5), SMBA (12.3), acetophenone (7.3). Conversions were calculated by comparing the areas of starting material and product, corrected by their response factor.

C18 Chiral RP-HPLC: : Waters XBridge BEH C18 Column Column (130Å, 3.5 µm, 2.1 mm X 150 mm); injection volume 2-20 µL at ambient temperature. Flow rate 0.8 mL/min, elution at 45 °C . Elution (A: 0.1% TFA in water, B: 0.1% TFA in acetonitrile): Retention times (265 nm): 0-5 min (50% to 100% A); 5-6.6 min (100% A); 6.6-10min (95% A). Retention times in min (amine Fmoc-derivatised): tetrahydrofuran-3-amine (5.220 min), Methyl benzyl amine(6.443 min), Butylamine (6.3 min).

Chiral RP-HPLC: Phenomenex Lux Cellulose-2 chiral column (5 µm, 44.6 x 250 mm); injection volume 2-20 µL, at ambient temperature with a flow rate of 1 mL/min, with the following isocratic method (A: 0.1% TFA in water, B: 0.1% TFA in acetonitrile) 40% A 60% B. Retention times (265 nm): (*S*)-tetrahydrofuran-3-amine (11.3), ®-tetrahydrofuran-3-amine (12.3), (S)-Butylamine (13.003 min), (R)-Butanamine (11.893 min). Conversions were calculated from a calibration curve of authentic standards (following the formation of product).

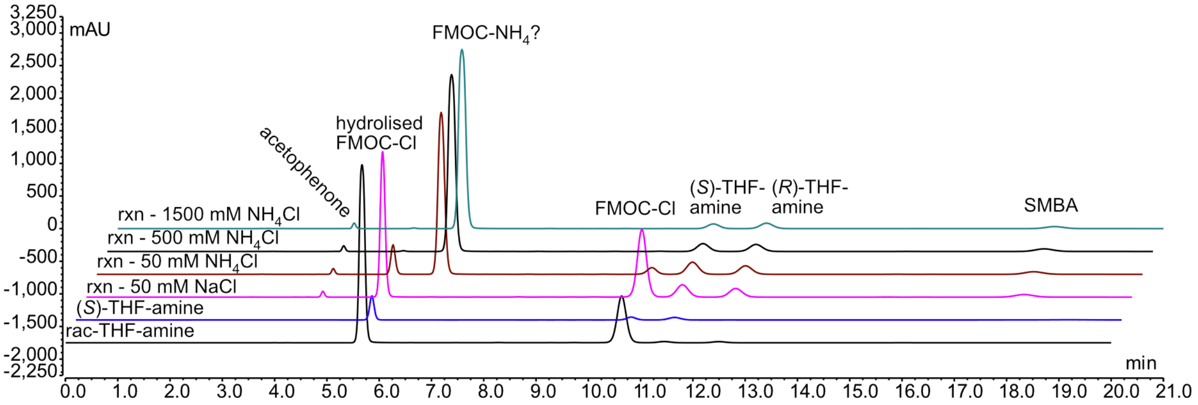

Figure S1: Representative chiral RP-HPLC chromatograms showing the complete consumption of FMOC in the presence of ammonium chloride, as well as the putative formation of $FMOC\text{-}NH_4$.
